# A Case of Embolic Spread of Fusobacterium necrophorum From Presumed Pharyngitis

**DOI:** 10.7759/cureus.18865

**Published:** 2021-10-18

**Authors:** Umar N Said, Khalid A Al-Hashimi

**Affiliations:** 1 Trauma and Orthopaedics, Huddersfield Royal Infirmary, Huddersfield, GBR; 2 Vascular Surgery, Royal Shrewsbury Hospital, Shrewsbury, GBR

**Keywords:** pharyngitis, sepsis, thrombophlebitis, lemierre's syndrome, fusobacterium necrophorum

## Abstract

*Fusobacterium necrophorum* is the most common pathogen isolated in individuals diagnosed with the rare and life-threatening illness known as Lemierre’s syndrome. Lemierre’s syndrome commonly involves a triad of infection in the oropharyngeal region, thrombophlebitis of the internal jugular vein, and distant metastases of said infection. Our case involves an embolic spread of *F. necrophorum* to the lungs, which was presumed to have originated in the pharynx, in the absence of internal jugular vein thrombosis. The clinical course of the patient was further complicated by an initial diagnosis of community-acquired pneumonia, severe sepsis, and disseminated intravascular coagulation. After suitable input from the multi-disciplinary team and adequate antibiotic therapy, the patient demonstrated a positive outcome with complete recovery to her baseline.

## Introduction

*Fusobacterium necrophorum* is a Gram-negative bacillus that can cause serious systemic infections. Lemierre’s syndrome is one such complication that can occur as a result of *F. necrophorum* infection. This syndrome was first reported in 1936 by Andre Lemierre [1].

The exact definition of Lemierre’s syndrome differs in the literature, however, the common theme is an oropharyngeal infection commonly with *F. necrophorum*, leading to thrombophlebitis of the internal jugular vein and dissemination to remote sites via septic emboli. Atypical presentations can also occur involving other pathogens, which can make diagnosis more challenging [2]⁠. The classical diagnosis of Lemierre’s syndrome involves meeting three criteria - a primary site of infection in the head or neck, thrombophlebitis of the internal jugular vein or metastatic septic emboli, and the isolation of *F. necrophorum* from blood cultures [3]⁠.

Disseminated cases of infection with *F. necrophorum* are rarely seen in clinical practice today with an incidence of 3 per 1 million per year. Mortality rates in the pre-antibiotic era reached 90%, while currently, mortality rates range from 0 to 18%, thus highlighting the need for rapid diagnosis and treatment [1,4]⁠. We report an atypical case of a 39-year-old female presenting with a pulmonary embolic spread of *F. necrophorum* from presumed pharyngitis, in the absence of internal jugular vein thrombophlebitis.

## Case presentation

A 39-year-old female with a background of IV recreational drug usage presented to the accident and ED feeling generally unwell with a one-week history of a sore throat and hoarse voice. On examination, she was clinically septic with a temperature of 38.2 degrees celsius, tachycardic with a heart rate of 100, and hypotensive with a blood pressure of 83/40. Her oxygen saturation on room air was 96%. Her blood tests on admission revealed a low hemoglobin level of 82 g/L, low platelet levels of 2 x 10*9/L, normal white cell count of 6.50 x 10*9/L, International Normalised Ratio (INR) of 1.5, C-reactive protein of 173 mg/L, and urea 18.5 mmol/L. The remaining blood profiles were unremarkable. Viral serology revealed the patient was positive for Hepatitis C antibodies, HIV was not detected.

**Figure 1:**
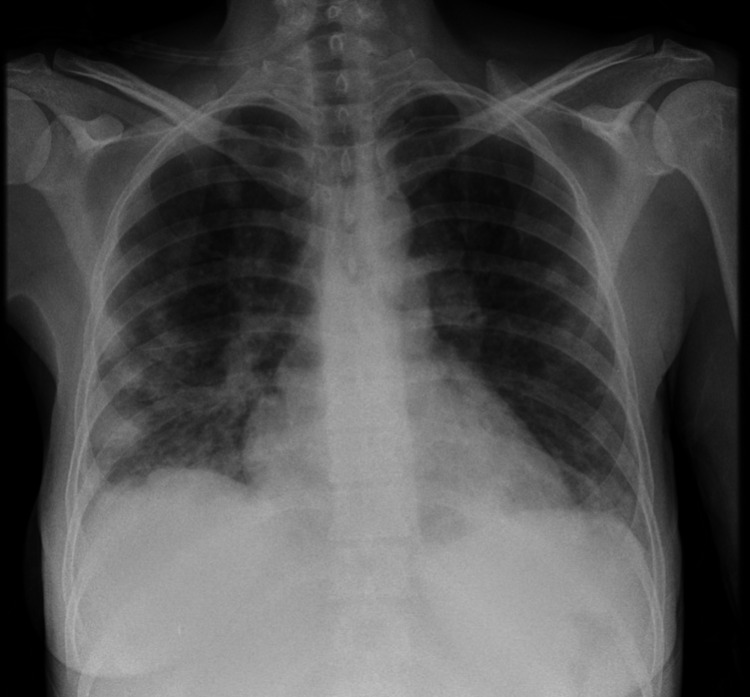
Chest X-ray on presentation. Chest X-ray taken on presentation at the Accident and ED showing focal consolidation in both lung fields in keeping with severe community-acquired pneumonia.

The patient was commenced on treatment in the accident and ED comprising of IV fluids and broad-spectrum antibiotic cover with cefotaxime and clarithromycin. These specific antibiotics were given due to chest X-ray changes on admission, demonstrating infective changes consistent with severe community-acquired pneumonia (Figure 1). The severe thrombocytopenia was discussed with a consultant hematologist who advised results were likely due to disseminated intravascular coagulation as a result of severe sepsis. The advice given was to maintain platelets above 20 x 10*9/L. The patient was transferred from the accident and ED to the acute medical unit for administration of RBCs and platelets, further antibiotics to treat presumed severe community-acquired pneumonia, and fluid resuscitation.

**Figure 2:**
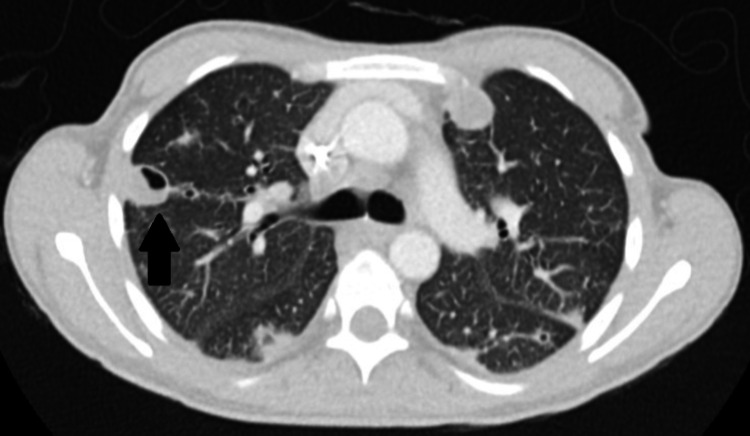
Chest CT with contrast. Chest CT scan demonstrating septic emboli (arrow).

**Figure 3:**
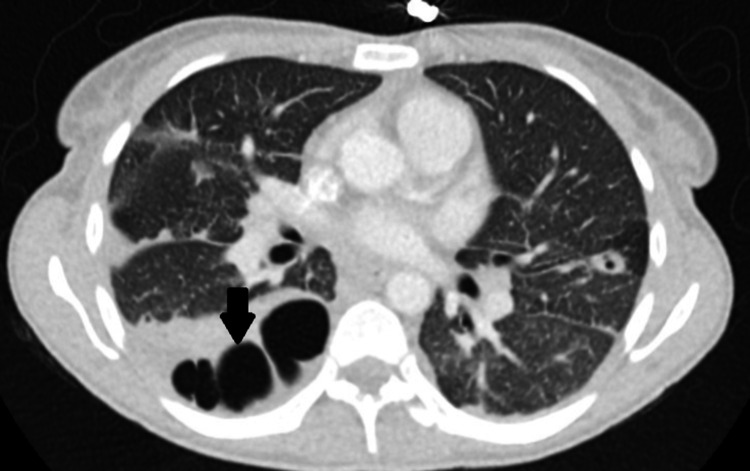
Chest CT with contrast. Chest CT scan demonstrating multiple lung cavitations (arrow).

Within 24 hours of admission to the acute medical unit, the patient deteriorated further. Her observations were 39.5 degrees Celsius, heart rate to 109, blood pressure 99/40, oxygen saturation of 95% on room air, and a respiratory rate of 25. Due to the severity of her clinical condition and her rapid deterioration despite optimal treatment, the patient was transferred to the ICU. A CT scan of the thorax revealed bilateral septic emboli and cavitations (Figures 2-3). A cardiothoracic surgeon was consulted as advised by the radiologist reporting the CT scan in regards to the cavitations in the lung. They advised that no surgical management was indicated. Blood cultures taken on admission grew isolated *F. necrophorum* and after consultation with a microbiologist, metronidazole was added to the antibiotic regime on day 3 of admission.

A CT scan of the neck interestingly demonstrated no evidence of internal jugular vein thrombophlebitis. A trans-thoracic ECG was performed which demonstrated no evidence of endocarditis. After a total of 17 days in the ICU, the patient was stepped down to a ward as she was no longer reliant on vasopressors or supplemental oxygen. In the ward, her antibiotic regime was changed to IV amoxicillin and oral metronidazole. The patient was then discharged after a total of 62 days as an inpatient on a four-week course of oral amoxicillin and metronidazole to be followed in a respiratory outpatient clinic.

**Figure 4:**
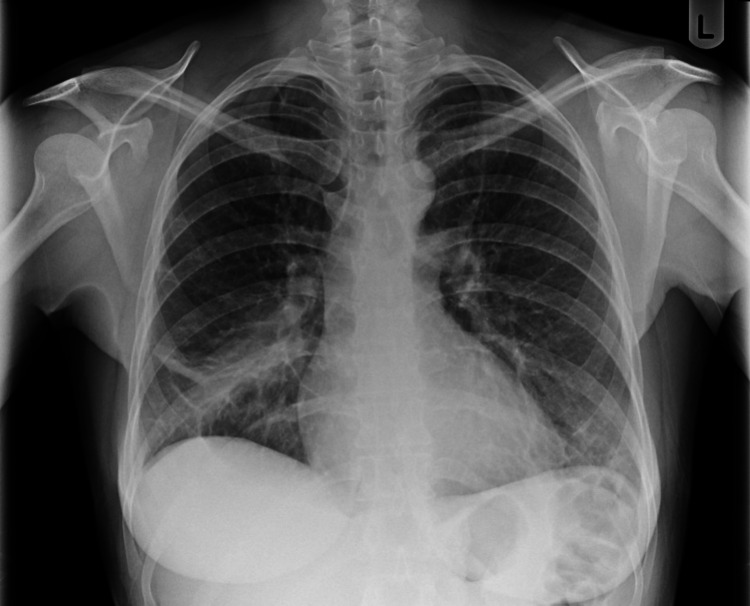
Chest X-ray one month after discharge. Chest X-ray taken one month after discharge from hospital demonstrating only partial improvement in consolidation.

**Figure 5:**
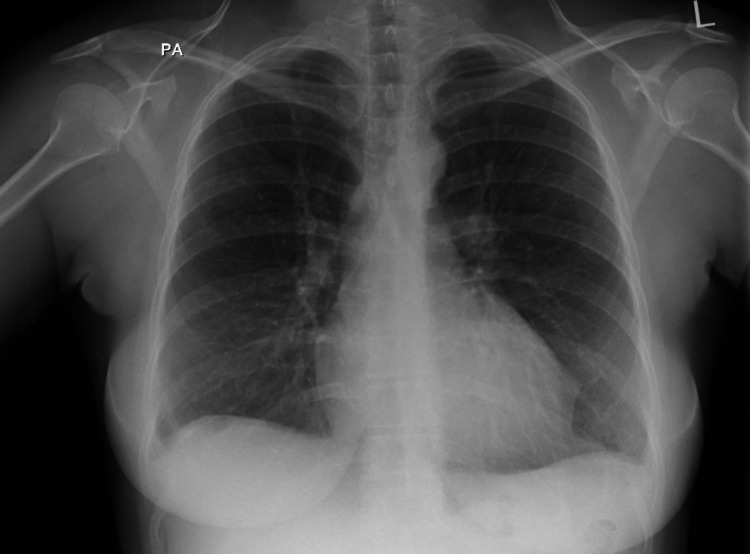
Chest X-ray three months after discharge. Chest X-ray taken three months after discharge from hospital demonstrating complete resolution of changes demonstrated earlier.

The patient was followed up in a respiratory clinic one month after being discharged. Chest X-rays taken at that time (Figure 4) demonstrated only a partial improvement and as a result, she was followed up in another clinic two months later. This final chest X-ray (Figure 5) demonstrated complete resolution of the consolidation in the right mid zone and left lower zone. She was then discharged from the care of the respiratory team.

## Discussion

While thrombophlebitis of the internal jugular vein is classical of Lemierre’s syndrome, it is not necessarily always present as demonstrated in this case. Literature also reports internal jugular vein thrombosis to only be present in 26-45% of patients diagnosed with Lemierre’s syndrome [3,5]⁠. The prodrome of a sore throat in this patient is highly suggestive of an oropharyngeal infection which then most likely resulted in sepsis with embolic spread to the lungs via a hematogenous route. The one-week history of a sore throat that the patient gave on initial presentation was likely disregarded by the initial medical team.

Lemierre’s syndrome remains a rare disease, with some papers reporting an incidence of only one case per million of the population per year [6]⁠. The diagnosis of Lemierre’s syndrome can prove difficult due to the rarity of the disorder, and also the fact that it often manifests initially as much more common conditions, including viral pharyngitis, bacterial or aspiration pneumonia, and staphylococcal endocarditis [7]⁠. In our case, the patient was being treated for severe community-acquired pneumonia until blood cultures confirmed growth of *F. necrophorum*, at which stage the diagnosis was reconsidered. Thrombocytopenia is commonly seen in patients diagnosed with Lemierre’s syndrome. Clinically significant disseminated intravascular coagulation as seen in our case is relatively uncommon being reported in only 3-9% of cases [5,8].⁠

A CT scan of the neck at presentation demonstrated no evidence of internal jugular vein thrombosis. A subsequent CT scan of the thorax demonstrated pulmonary involvement with septic emboli and cavitations. Pulmonary manifestations of Lemierre’s syndrome are extremely common occurring in up to 97% of cases [7]⁠. Input from hematology, microbiology, intensive care, cardiothoracic and respiratory teams allowed us to treat our patient optimally, ensuring she received adequate antibiotic therapy and follow-up. The patient made a full recovery and was discharged from the care of the respiratory team seven months after initial hospitalization. The patient was also offered support in regards to her drug usage but she declined any intervention offered to her.

## Conclusions

Lemierre’s syndrome is rarely seen in the current era. This case illustrates the importance of blood cultures and the consideration of a wider microbiological differential in light of the clinical history. The presence of a sore throat as a prodrome to respiratory symptoms should raise suspicion for atypical diagnoses and prompt rapid initiation of treatment with antibiotics given the high mortality rate associated with Lemierre’s syndrome. The absence of internal jugular vein thrombosis, but the presence of severe disseminated intravascular coagulation, in this case, demonstrates some of the complications which may manifest in Lemierre’s syndrome.

## References

[1] Lemierre A (1936). On certain septicæmias due to anaerobic organisms. Lancet.

[2] Hagelskjaer Kristensen L, Prag J (2000). Human necrobacillosis, with emphasis on Lemierre's syndrome. Clin Infect Dis.

[3] Sinave CP, Hardy GJ, Fardy PW (1989). The Lemierre syndrome: suppurative thrombophlebitis of the internal jugular vein secondary to oropharyngeal infection. Medicine (Baltimore).

[4] Eilbert W, Singla N (2013). Lemierre's syndrome. Int J Emerg Med.

[5] Moreno S, García Altozano J, Pinilla B, López JC, de Quirós B, Ortega A, Bouza E (1989). Lemierre's disease: postanginal bacteremia and pulmonary involvement caused by Fusobacterium necrophorum. Rev Infect Dis.

[6] Hagelskjaer LH, Prag J, Malczynski J, Kristensen JH (1998). Incidence and clinical epidemiology of necrobacillosis, including Lemierre's syndrome, in Denmark 1990-1995. Eur J Clin Microbiol Infect Dis.

[7] Riordan T, Wilson M (2004). Lemierre's syndrome: more than a historical curiosa. Postgrad Med J.

[8] Eykyn SJ (1989). Necrobacillosis. Scand J Infect Dis Suppl.

